# Therapeutic Effects of Tangshen Formula on Diabetic Nephropathy in Rats

**DOI:** 10.1371/journal.pone.0147693

**Published:** 2016-01-25

**Authors:** TingTing Zhao, SiFan Sun, HaoJun Zhang, XiaoRu Huang, MeiHua Yan, Xi Dong, YuMin Wen, Hua Wang, Hui Yao Lan, Ping Li

**Affiliations:** 1 Beijing Key Lab for Immune-Mediated Inflammatory Diseases, Institute of Clinical Medical Sciences, China-Japan Friendship Hospital, Beijing, China; 2 Department of Medicine and Therapeutics, and Li Ka Shing Institute of Health Sciences, The Chinese University of Hong Kong, Hong Kong SAR, and Shenzhen Research Institute, The Chinese University of Hong Kong, Shenzhen, China; University of Utah School of Medicine, UNITED STATES

## Abstract

**Objective:**

Inflammation and fibrosis are essential promoters in the pathogenesis of diabetic nephropathy (DN) in type 2 diabetes. The present study examined the anti-inflammation and anti-fibrosis effect of Tangshen Formula (TSF), a traditional Chinese medicine, on DN.

**Research Design and Methods:**

Protective role of TSF in DN was examined in a rat model of type 2 DN that was established by high-fat diet-fed and low-dose-streptozotocin injection. TSF was suspended in 0.5% CMC-Na solution and delivered by oral gavage at a dosage of 1.67g/Kg body weight/day. The therapeutic effects and mechanisms of TSF on diabetic kidney injury were examined.

**Results:**

We found that TSF treatment for 20 weeks attenuated DN by significantly inhibiting urinary excretion of albumin and renal histological injuries. These beneficial effects were associated with an inactivation of NF-κB signaling, thereby blocking the upregulation of pro-inflammatory cytokines (IL-1β, TNFα), chemokine (MCP-1), and macrophage infiltration in the TSF-treated rats with type 2 DN. In addition, TSF treatment also inactivated TGF-β/Smad3 signaling and therefore suppressed renal fibrosis including expressions of fibronectin, collagen I, and collagen IV. Further studies revealed that the inhibitory effect of TSF on TGF-β/Smad3 and NF-κB signaling in DN was associated with inhibition of Smurf2-dependent ubiquitin degradation of Smad7.

**Conclusions:**

The present study reveals that TSF has therapeutic potential for type 2 DN in rats. Blockade of NF-κB-driven renal inflammation and TGF-β/Smad3-mediated renal fibrosis by preventing the Smurf2-mediated Smad7 degradation pathway may be mechanisms through which TSF inhibits type 2 DN.

## Introduction

Diabetic nephropathy (DN) is one of the major microvascular complications of diabetes. It is characterized by glomerular hypertrophy, excessive deposition of extracellular matrix in the mesangium and tubulointerstitium with a slow progression to renal fibrosis accompanied by the development of microalbuminuria and a decline of glomerular filtration rate (GFR) [1, 2]. Emerging studies consistently indicate that inflammation is an important mechanism that leads to the development and progression of DN [3]. Macrophages and many inflammatory mediators including TNFα, MCP-1, ICAM-1, IL-1, IL-6, and IL-18 have been shown to be significantly increased in the diabetic kidney in patients with type 2 DN and correlated strongly with the progression of DN as indicated by proteinuria, interstitial fibrosis, and GFR decline [4, 5].

TGF-β/Smad signaling has been recognized as one of the key pathways in the development of inflammation and fibrosis in many kidney diseases including DN [6, 7]. It is well established that TGF-1 binds to its receptors, and then activates its downstream signaling molecules, Smad2 and Smad3, to mediate fibrosis [8, 9]. Overexpression of TGF-β1 promotes extracellular matrix production and at the same time inhibits its degradation, resulting in progressive renal fibrosis [10]. Activation of TGF-β/Smad3 signaling also causes a degradation of Smad7, an inhibitory Smad that negatively regulates TGF- signaling *via* the ubiquitin-proteasome degradation mechanism [8, 9, 11]. In addition, Smad7 can negatively regulate NF-κB signaling *via* induction of IκBα, an inhibitor of NF-κB, thereby blocking IκBα from degradation and preventing the activation of inflammation driven by NF-κB signaling in *vivo* and in *vitro* [12, 13].

Although a significant progress has been made in our understanding of the pathogenesis of DN, treatment for DN remains ineffective. Therefore, it is an urgent need for searching and developing strategies that can prevent and effectively attenuate the DN progression. In China, traditional Chinese medicine (TCM) is widely used for the treatment of diabetes and its complications, and has become a promising source of new therapeutic agents for DN [14–16]. Tangshen Formula (TSF) is formulated based on the individual property of TCM for the treatment of diabetic kidney disease. We have reported that treatment with TSF significantly improved estimated GFR (eGFR) in patients with diabetes mellitus who have either microalbuminuria or macroalbuminuria [17], but the underlying mechanisms of TSF on DN remain largely unexplored. In the present study, we investigated the anti-inflammatory and anti-fibrotic effects of TSF on DN in a type 2 diabetic nephropathy in rats.

## Materials and Methods

### Herbal formulation and components

TSF was extracted from seven natural herbs: astragalus root (*Astragalus membranaceus* (Fisch.) Bge.), burning bush twig (*Euonymus alatus* (Thunb.) Sieb.), rehmannia root (*Rehmanniaglutinosa*Libosch.), bitter orange (*Citrus aurantium* L.), cornus fruit (*Cornus officinalis* Sieb. et Zuce), rhubarb root and rhizome (*Rheum palmatum* L.), notoginseng root (*Panaxnotoginseng* (Burk.) F.H. Chen) in the ratio of 10:5:4:3.4:3:2:1 (W/W), respectively on a dry-weight products. The TSF powder was prepared and standardized by an established company (Jiangyin Tianjiang Pharmaceutical, Jiangsu, China, http://www.tianjiang.com) which is well recognized in China as the high quality control standards.

Quality control for the raw materials of herbs and the final powder was performed according to established guidelines in the *Pharmacopoeia of The People’s Republic of China 2010* [18]. Chemical composition of TSF was measured using high-performance liquid chromatography/mass spectrometry (HPLC/MS). Nine most representative compounds were identified in TSF, including: sweroside from *Cornus officinalis* Sieb. et Zuce, rhapontigenin from *Rheum palmatum* L., isomucronulatol-7, 2'-di-glucoside from *Astragalus membranaceus* (Fisch.) Bge., naringin from *Citrus Aurantium* L., isonaringinfrom *Citrus Aurantium* L., melittoside from *Rehmannia glutinosa* Libosch, ginsenoside Rg1 from *Panax Notoginseng* (Burk) F.H. Chen, morroniside from *Cornus officinalis* Sieb. et Zuce, ginsenoside Rb1 from *Panax Notoginseng* (Burk) F.H. Chen.

### Animals and experimental design

Thirty-five male Wistar rats (8-weeksold, 200–250grams) were purchased from Beijing HFK Bio-Technology (Beijing, China, Certificate No. SCXK 2002–0010) and randomly divided into normal control group (n = 13) and diabetic group (n = 22). The diabetic rat model was induced according to an established protocol [19]. Briefly, to induce type 2 diabetes, rats were fed with a high-fat diet (38% fat, 12% protein, and 50% carbohydrate) for 5 weeks, whereas, normal control animals received a standard rat chow. After 5 weeks, 4 rats in each group were randomly chosen to receive hyperinsulinemic-euglycemic clamping [20]. All rats in the diabetic group then received a single intraperitoneal injection of a low-dose streptozotocin (STZ, 25 mg/kg, Sigma-Aldrich, St. Louis, MO, USA), while normal control animals received an equivalent dose of citrate buffer solution (pH 4.4). Development of hyperglycemia was confirmed by measuring fasting blood glucose at 72 hours after injection. Rats with fasting blood glucose level above 11.1μmmol/L (One Touch Ultraglucose meter, Life Scan, Milpitas, CA USA) were then randomly divided into 2 subgroups: vehicle-treated or TSF-treated animals (n = 9 each). TSF treatment was initiated on the third day after STZ injection and continued for 20 weeks. TSF was suspended in 0.5% CMC-Na solution and delivered by oral gavage at a dosage of 1.67g/Kg body weight/day. Rats in normal group received the same volume of 0.5% CMC-Na solution without TSF.

All animals were housed at a temperature of 20–25°C, humidity of 65–69%, and were subjected to a 12-hour light/dark cycle with free access to food and tap water. Rats were kept in individual metabolic cages (Fengshi Inc., Suzhou, JS, China) for a 24-hoururinary collection in a 4-week interval to measure urine volume. Urinary levels of albumin was measured by a competitive ELISA method according to the manufacturer’s instructions (Exocell, Philadelphia, PA, USA) and presented as the albumin to creatinine ratio (ACR). Body weight was measured every two weeks. Blood glucose was measured every 4 weeks by tail-vein blood sampling with One Touch Ultra blood glucose monitoring system (LifeScan, Milpitas, CA USA). Rats were sacrificed and aortic blood was collected without anticoagulant and centrifuged at 3000 g/min and 4°C for 15 min. Serum was separated and creatinine was measured using a CD-1600C Shematology analyzer (Abbott Labs, USA).

Experimental procedures were approved by the Ethics Committee of China-Japan Friendship Hospital, Institute of Clinical Medical science (No. 2012-A04) and performed in accordance with the The National Academies *Guiding Principles* for *the Care and Use of Laboratory Animals*, 8^th^ edition.

### Histology and immunohistochemistry

Rats treated with or without TSF were sacrificed at 20 weeks after treatment. Kidney tissues were fixed in 10% phosphate buffered formalin solution, embedded in paraffin, and then sectioned into 3-μm thicknesses and stained with Masson’s trichrome and periodic acid-Schiff (PAS). Glomerulosclerosis was defined as the percentage of ECM deposition and mesangial expansion and evaluated at 400× power for 20 cortical fields. Tubulointerstitial damage was assessed based on the established method and scoring system [14]. Briefly, the percentage of tubulointerstitial damage including tubular atrophy and dilation, formation of casts, as well as interstitial mononuclear cell and extracellular matrix accumulation (interstitial volume) was scored in 10-cortical fields (200×). The scoring system was as follows: 1 = less than 10%; 2 = 10–25%; 3 = 26–50%, 4 = 51–75%, 5 = 76–95%, 6 = more than 95%.

A microwave-based antigen retrieval method was applied to conduct immunohistochemistry on the paraffin sections [21]. Antibodies used included: primary antibodies against fibronectin, phosphorylated Smad2/3 (p-Smad2/3), TNFα, IL-1β, and TGF-β1 (all purchased from Santa Cruz Biotechnology, Dallas, TX, USA), collagens I and IV (SouthernBiotech, Birmingham, AL, USA), MCP-1 (eBioscience, San Diego, CA, USA), phosphorylated-NF-κB/p65 (p-p65; Cell Signaling Technology, Danvers, MA, USA) and ED-1 (AbDSerotec, Kidlington, UK). After being immuno stained, sections were developed with diaminobenzidine to produce a brown product. The sections were then counterstained with hematoxylin. Deposition of collagen I and fibronectin was measured using the quantitative Image Analysis System (Image-Pro Plus v 6.0, Media Cybernetics, Warrendale, PA, USA). Briefly, in both glomeruli and tubulointerstitium, 10 random fields were outlined and positive staining patterns were identified under 200× power. Then, the percentage of positive area in the examined field was measured. Accumulation of collagen IV in 20 random glomeruli under 400 × power was analyzed as above. The arterial lumen space was excluded from the study. Numbers of p-Smad2/3^+^, p-p65^+^cells were counted in 20 random glomeruli and expressed as cells/glomerular cross-section, whereas positive cells in the tubulointerstitium were counted in 20 random fields for each sample under high-power fields (400×) by means of a 0.0625-mm^2^graticule fitted in the eyepiece of the microscope, and expressed as cells per mm^2^ [14]. ED-1^+^ macrophages were randomly counted in twenty fields of cortex per section under high-power fields (400×), and expressed as cells per mm^2^ [12]. All counting was performed on blinded slides. Data were expressed as the means ± SE.

### Extraction of RNA and RT-PCR analysis

Renal cortical tissues were collected by carefully removing the renal pelvis and medullar tissues and were frozen at -80°C for extraction of total RNA by Trizol reagent (Invitrogen, Life Technologies, Carlsbad, CA, USA). Template cDNA was prepared by using reverse transcriptase (Thermo Scientific, Lithuania). The genes of interest were analyzed by real-time PCR following the manufacturer’s instructions (ABI system, USA).

Primers used for detection of rat mRNAs were: collagen IV: forward 5′-GGCGGTGCACAGTCAGACCAT-3′ and reverse 5′-GGAATAGCCAATCCA CAGTGA-3′; collagen I: forward 5′-CACAAGCGTGCTGTAGGTGA-3′ and reverse 5′-TGCCGTGACCTCAAGATGTG-3′; fibronectin: forward 5′-GAGGAGGTCCAAATCGGTCATGTT-3′ and reverse 5′-AACTGTAAGGGCTCTTCGTCAATG-3′; TGF-β1: forward 5′-ACGTCAGACATTCGGGAAGCAGTG-3′ and reverse 5′- GCAAGGACCTTGCTGTACTGTGTG-3′; Smad3: forward 5′-CTCCTACTACGAGCTGAACCAG-3′ and reverse 5′-CTCATGCGGATGGTGCACATTC-3′; MCP-1 forward 5`-CACTGGCAAGATGATCCCAATG-3` and reverse 5`-CTTCTA CAGAAGTGCTTGAGGTGG-3`; TNFα forward 5`-GCACAGAAAGCATGATCCGAGTG-3` and reverse 5`-TTGGGAACTTCTCCTCCTTGTTGG-3`; IL-1β forward 5`-TCACTCATTGTGGCTGTGGAGAAG-3` and reverse 5`-CACACACTAGCAGGTCGTCATCAT-3`; β-actin: forward 5′-GAGACCTTCAACACCCAGCC-3′ and reverse 5′-GCGGGGCATCGGAACCGCTCA-3′. The ratio of mRNA expression was examined to house-keeping gene β-actin and expressed as the means ± SE.

### Western blot analysis

Proteins for Western blot analysis were extracted from the renal cortex by lysing with radioimmunoprecipitation assay (RIPA) buffer as described previously [22]. Antibodies used in this study included primary antibodies against fibronectin, TGF-β1, TGF-β receptor I (TβRI), phospho-TβRI (p-TβRI), TGF-β receptor II (TβRII), Smad ubiquitination regulatory factor 2 (Smurf2)and Smad7 (Santa Cruz Biotechnology, Santa Cruz, CA, USA), Smad3 (Zymed Laboratories, South San Francisco, CA, USA),p-p65, phospho-Smad3 (p-Smad3) and IκB-α (Cell Signaling Technology Inc., Danvers, MA, USA), collagen I and IV (Southern Biotech, Birmingham, AL, USA), and IRDyeTM800 conjugated secondary antibodies (Rockland Immunochemicals Inc., Gilbertsville, PA, USA). Signals were detected with Odyssey Infrared Imaging System (LI-COR Biosciences, Lincoln, NE, USA) and quantified with Image J program (National Institutes of Health, Bethesda, MD, USA). Ratio for the protein examined was normalized against β-actin and expressed as means± SE.

### Statistical analysis

Data collected from this study were expressed as means ± SE and analyzed using ANOVA, followed by post comparison using the Newman Keuls program from GraphPad Prism 5.0 (GraphPad Software, San Diego, CA, USA).

## Results

### TSF treatment attenuates proteinuria and histological damage in the diabetic kidney with type 2 diabetes in rats

**Fig 1:**
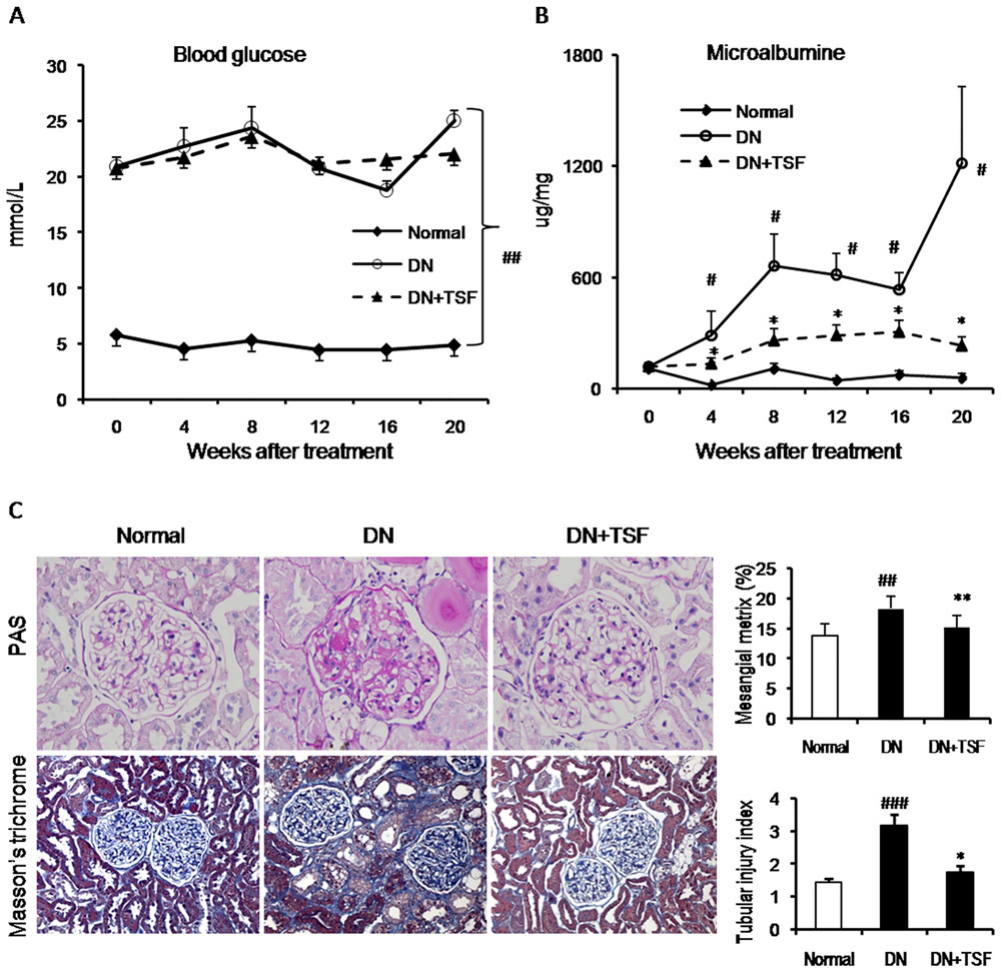
TSF attenuates renal injury in DN rats. (A) Blood glucose. Blood glucose levels in DN rats are significantly increased after STZ-injection and high-fat-diet induction and maintained at equal higher levels during the 20 weeks. TSF-treated rats exhibit no effect on blood glucose level. (B) Microalbuminuria. DN rats without TSF treatment show more severe microalbuminuria than those with TSF treatment over 4–20 weeks. (C) Histology (PAS staining, original magnification, × 400; Masson’s trichrome staining, original magnification, × 200) and semi-quantification of mesangial matrix and tubular injury. Data are expressed as means ± SE for each group of 9 rats. *P< 0.05, **P<0.01 TSF-treated group vs. DN group; ^#^*P*< 0.05, ^##^*P*< 0.01, ^###^*P*< 0.001 DN group vs. normal group.

After a low dose STZ injection, rats fed with the high-fat diet developed hyperglycemia at the first week (termed week 0) and maintained at high levels of blood glucose over the 20-weekstudyperiod (Fig 1A). Microalbuminuria in diabetic rats increased markedly at the 4th week and peaked at week 20, which was significantly reduced in the diabetic rats treated with TSF (Fig 1B). Compared to the age-matched normal rats, diabetic rats also developed a significant weight loss from weeks 4 to 20 (S1A Fig). Meanwhile, a significant increase in urine volume in diabetic rats was observed from the 8th week after STZ injection (S1B Fig). Interestingly, compared to normal rats, levels of serum creatinine in diabetic rats were lower, which was not altered by TSF treatment (S1C Fig). No effects on blood glucose level, body weight and urine volume were observed (Fig 1A and S1B and S1C Fig).

Histologically, kidneys from diabetic rats showed a moderate mesangial matrix expansion, thickening of the glomerular basement membrane, tubular atrophy, and extracellular matrix deposition (Fig 1C). Treatment with TSF for 20 weeks significantly inhibited these histological injuries (Fig 1C).

### TSF treatment inhibits renal inflammation and fibrosis in the diabetic kidney with type 2 diabetes in rats

**Fig 2:**
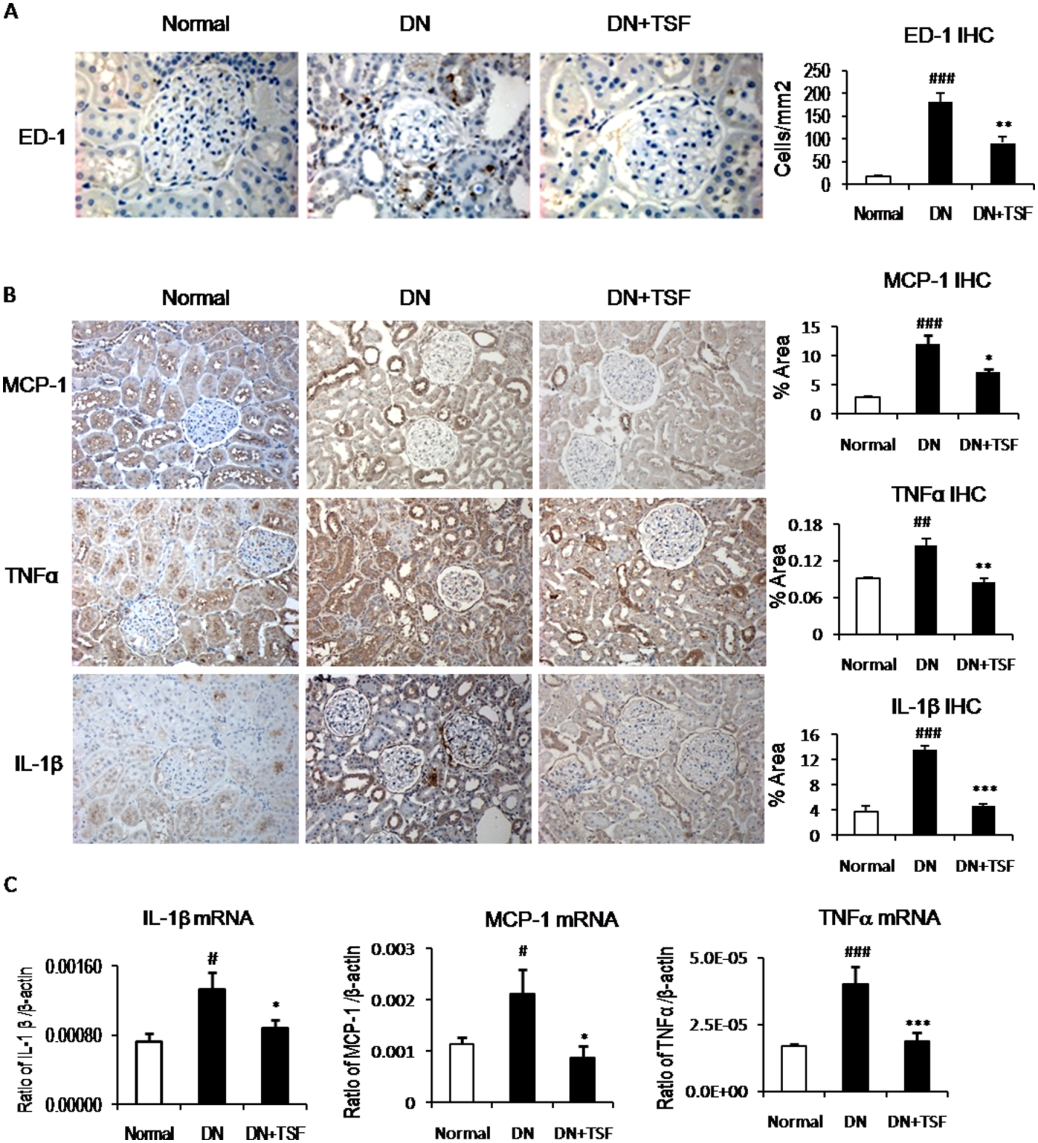
TSF inhibits renal inflammation in DN rats. (A) Infiltration of monocytes/macrophages. ED-1-positive cells were detected by immunohistochemistry and calculated per high-power field (400 ×). (B) Immunohistochemistry and semi-quantitative analysis for MCP-1, TNFα, and IL-1β. (C) Real-time PCR for IL-1β, MCP-1, and TNFα. Original magnification, × 200. TSF-treated rats exhibit a significant inhibition of renal inflammation including ED-1, MCP-1, IL-1β, and TNFα. Data are expressed as means ± SE for each group of 9 rats. *P<0.05, **P<0.01 TSF-treated group vs. DN group; ^#^*P*<0.05, ^##^*P*<0.01, ^###^*P*<0.001 DN group *vs*. normal group.

**Fig 3:**
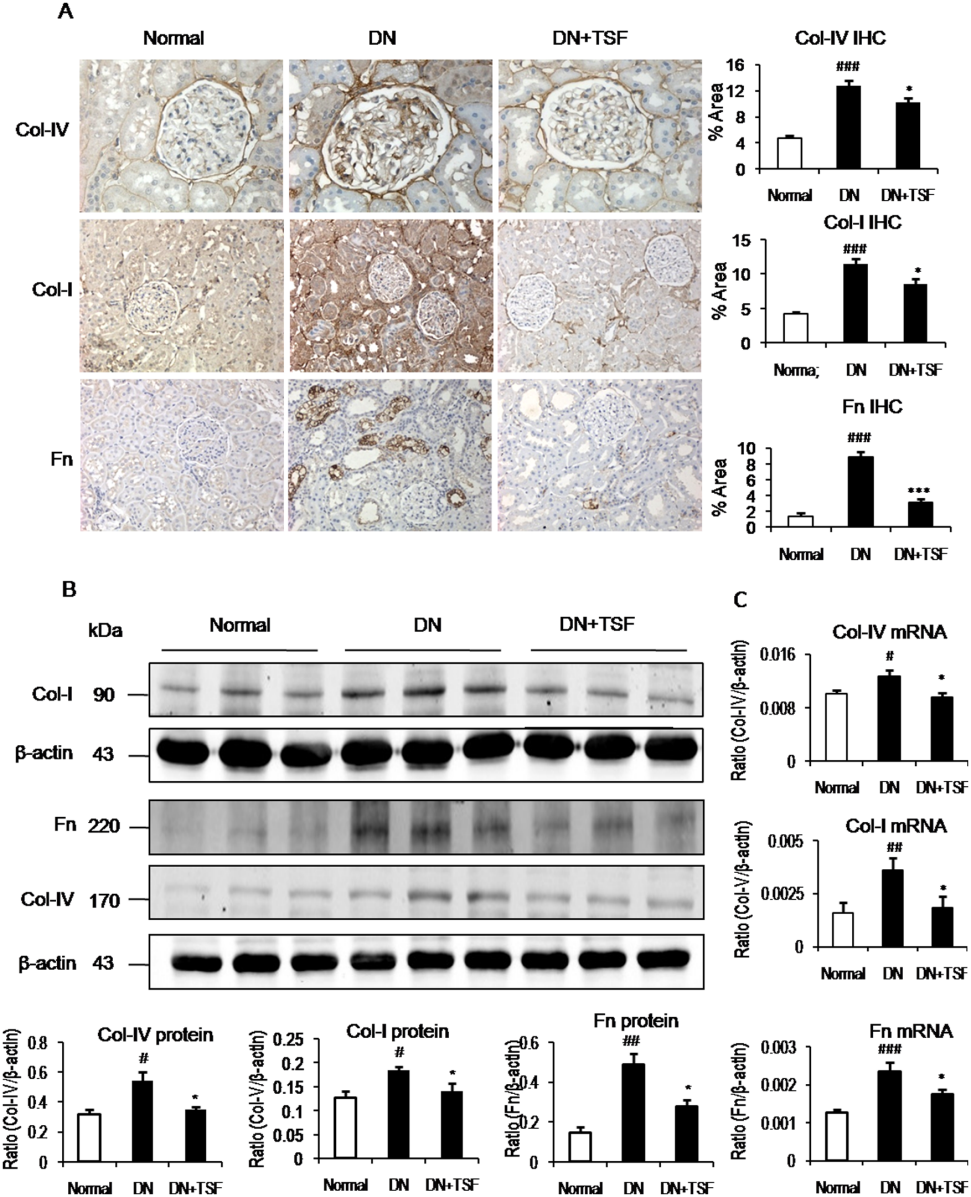
TSF inhibits renal fibrosis in DN rats. (A) Immunohistochemistry (IHC) and quantitative analysis of collagen IV (Col-IV, original magnification, × 400), collagen I (Col-I, original magnification, × 200), and fibronectin (Fn, original magnification, × 200). (B) Western blot and quantitative analysis of collagen I, collagen IV, and fibronectin. (C) Real-time PCR of collagen I, collagen IV, and fibronectin. Results show that compared with the normal rats, renal fibrosisis markedly enhanced in DN rats. However, in TSF-treated DN rats renal fibrosis is inhibited as demonstrated by Western blot analysis, IHC at the protein level and real-time PCR at the mRNA level. Data are expressed as means ± SE for each group of 9 rats. *P<0.05, **P<0.01 TSF-treated group vs. DN group; ^#^*P*< 0.05, ^##^*P*<0.01, ^###^*P*<0.001 DN group *vs* normal group.

We then examined the therapeutic effect of TSF on renal inflammation and fibrosis in diabetic rats. Immunohistochemistry and real time PCR revealed that rats with type 2 diabetes developed moderate renal inflammation, including many ED1^+^ macrophage infiltration and a significant upregulation of proinflammatory cytokines (IL-1β, TNFα) and macrophage chemotactic molecule-1 (MCP-1) at both mRNA and protein levels, which was attenuated by treatment with TSF (Fig 2). Similarly, a moderate renal fibrosis such as upregulation and accumulation of collagen I, collagen IV, and fibronectinin the diabetic kidney was largely reduced after treatment with TSF (Fig 3).

### Inhibition of TGF-β/Smad3 and NF-κB signaling pathways may be mechanisms by which TSF attenuates diabetic renal fibrosis and inflammation

**Fig 4:**
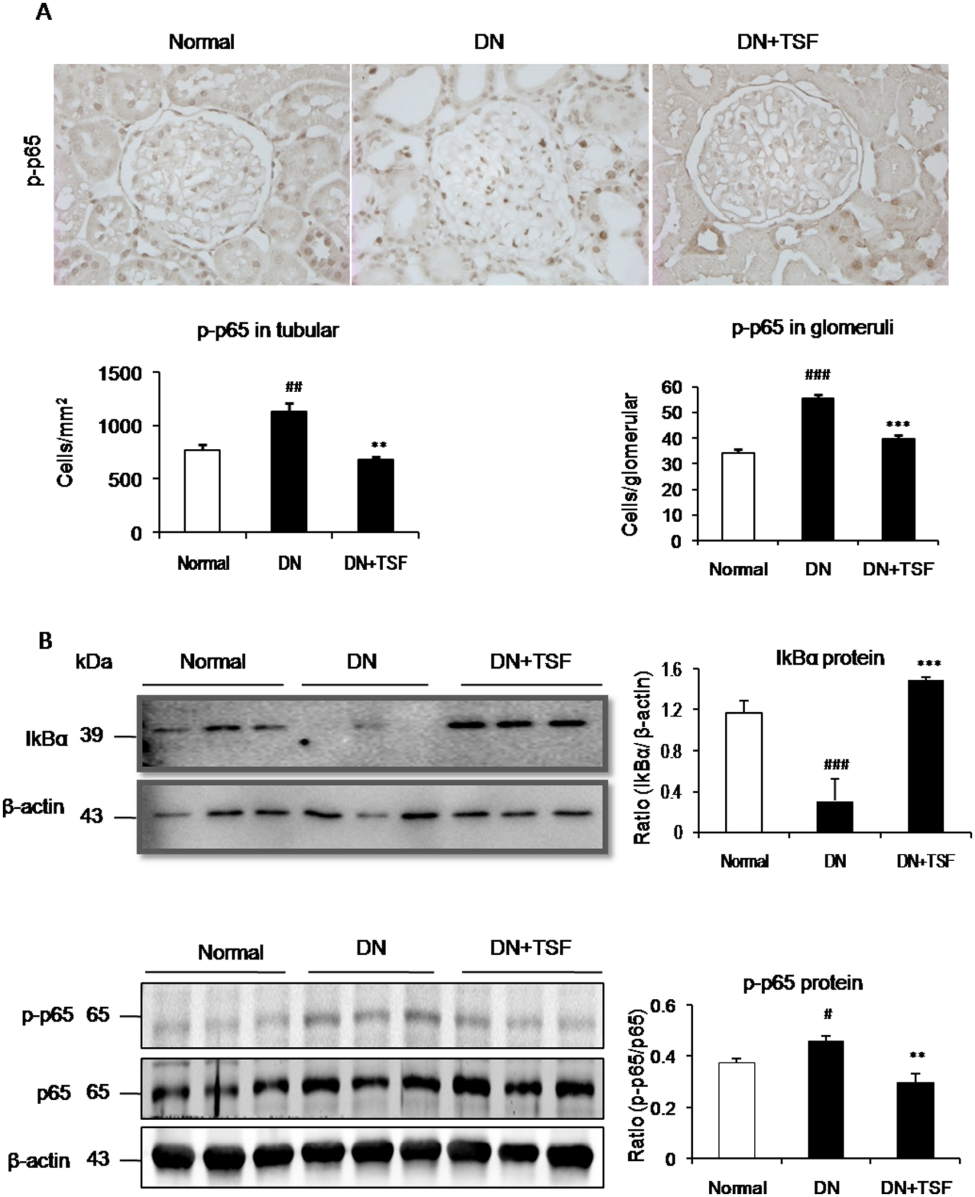
TSF blocks activation of the NF-κB signaling pathway in DN rats. (A) Immunohistochemistry and semi-quantitative analysis of phosphorylated NF-κB/p65 nuclear location in the glomeruli and tubulointerstitium. Original magnification, × 400. (B) Western blot and semi-quantitative analysis of renal phosphorylation of IκBα and NF-κB/p65 (p-p65). Data are expressed as means ± SE for each group of 9rats.*P<0.05, **P<0.01 TSF-treated group vs. DN group; ^#^*P*<0.05, ^##^*P*< 0.01, ^###^*P*<0.001 DN group *vs* normal group.

**Fig 5:**
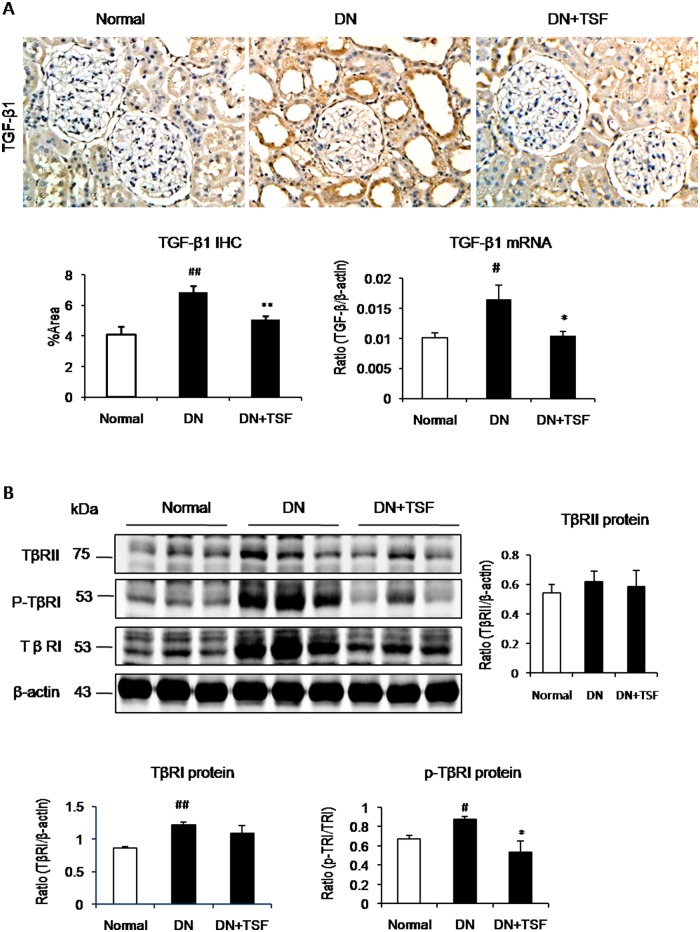
TSF treatment inhibits expression of the TGF-β1 and TGF-β receptors in DN rats. (A) Immunohistochemistry. Original magnification,× 200and real-time PCR for TGF-β1. (B) Western blot and semi-quantitative analysis of renal TGF-β receptor II (TβRII), phosphorylation of TGF-β receptor I (p-TβRI) and TβRI, respectively. A marked activation of TGF-β/TGF-β receptor signaling in the diabetic kidney is blocked by TSF treatment. Data are expressed as means ± SE for each group of 9 rats. *P<0.05, **P<0.01 TSF-treated group vs. DN group; ^#^*P*<0.05, ^##^*P*<0.01, ^###^*P*<0.001 DN group vs. normal group.

**Fig 6:**
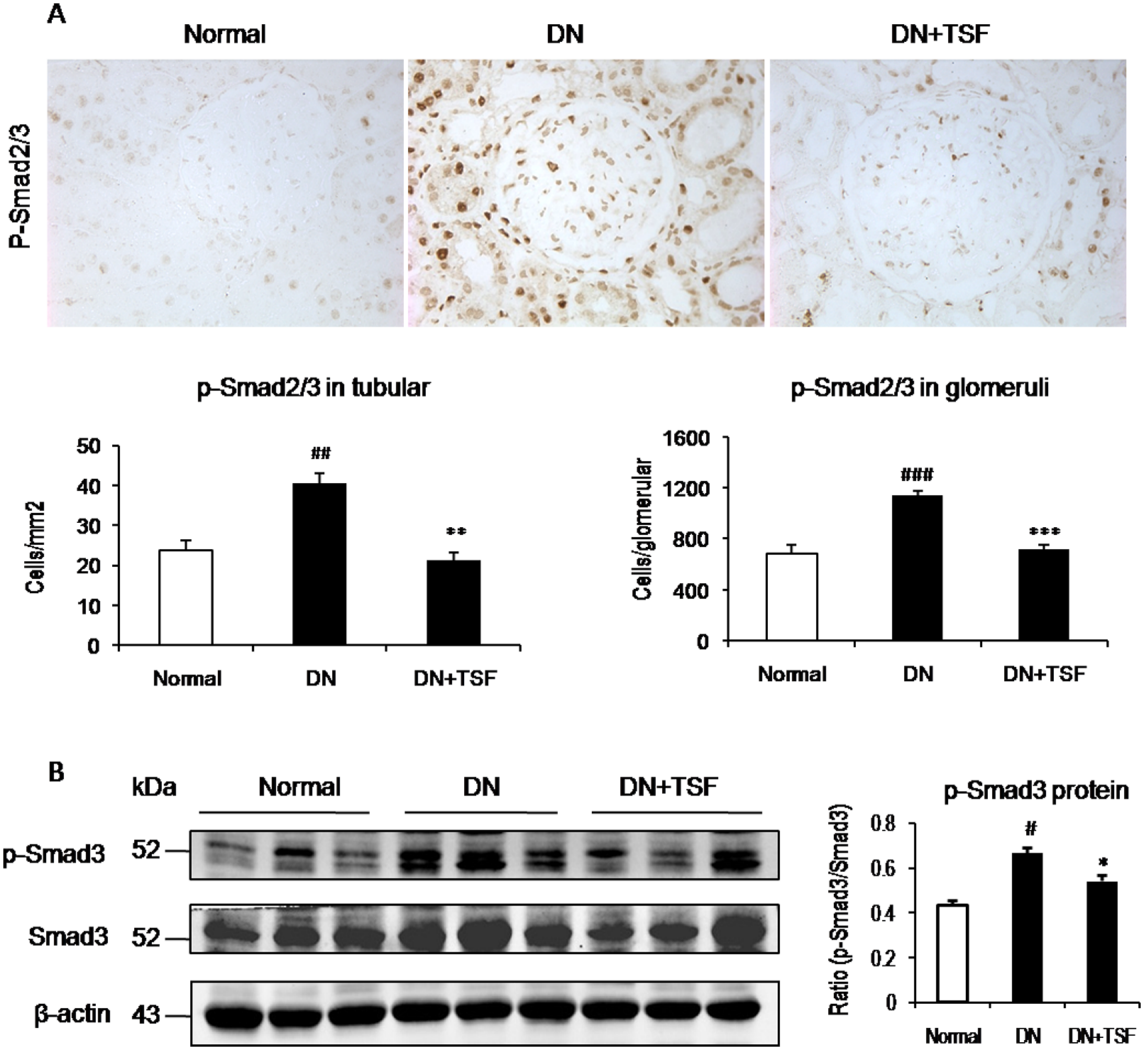
TSF blocks activation of Smad2/3 in DN rats. (A) Immunohistochemistry and semi-quantitative analysis of phosphorylated Smad2/3 nuclear location in the glomeruli and tubulointerstitium. Original magnification, × 400. (B) Western blot and semi-quantitative analysis of renal phosphorylation of Smad3 (p-Smad3) and Smad3. Data are expressed as means ± SE for each group of 9rats. *P<0.05, **P<0.01 TSF-treated group vs. DN group; ^#^*P*<0.05, ^##^*P*<0.01, ^###^*P*<0.001 DN group *vs* normal group.

It has been well documented that TGF-β/Smad signaling is a major pathway leading to renal fibrosis and the activation of NF-κB signaling plays a critical role in renal inflammation [23, 24]. We thus investigated the potential mechanisms by which TSF treatment attenuates diabetic renal fibrosis and inflammation by examining the TGF-β/Smad3 and NF-κB signaling pathways. Immunohistochemistry and western blot analysis showed that treatment with TSF blocked the activation of NF-κB signaling in the diabetic kidney by largely inhibiting IκBα degradation and phosphorylated NF-κB/p65 nucleated translocation (Fig 4). Further study also showed that treatment with TSF resulted in a marked suppression of the activation of TGF-β/Smad3 signaling by inhibiting expression of TGF-β1 and its receptor I (TβRI, p-TβRI), phosphorylation of Smad3 protein and its nuclear translocation in the diabetic kidney (Figs 5 and 6). Thus, results from this study revealed that treatment with TSF significantly suppressed diabetic kidney injury via mechanisms associated with NF-κB-driven renal inflammation and TGF-β/Smad3-mediated renal fibrosis.

### Enhanced renal Smad7 is an underlying mechanism required for the treatment of TSF on down-regulating of TGF-β/Smad-mediated renal fibrosis and NF-κB-driven inflammation in type 2 diabetes in rats

**Fig 7:**
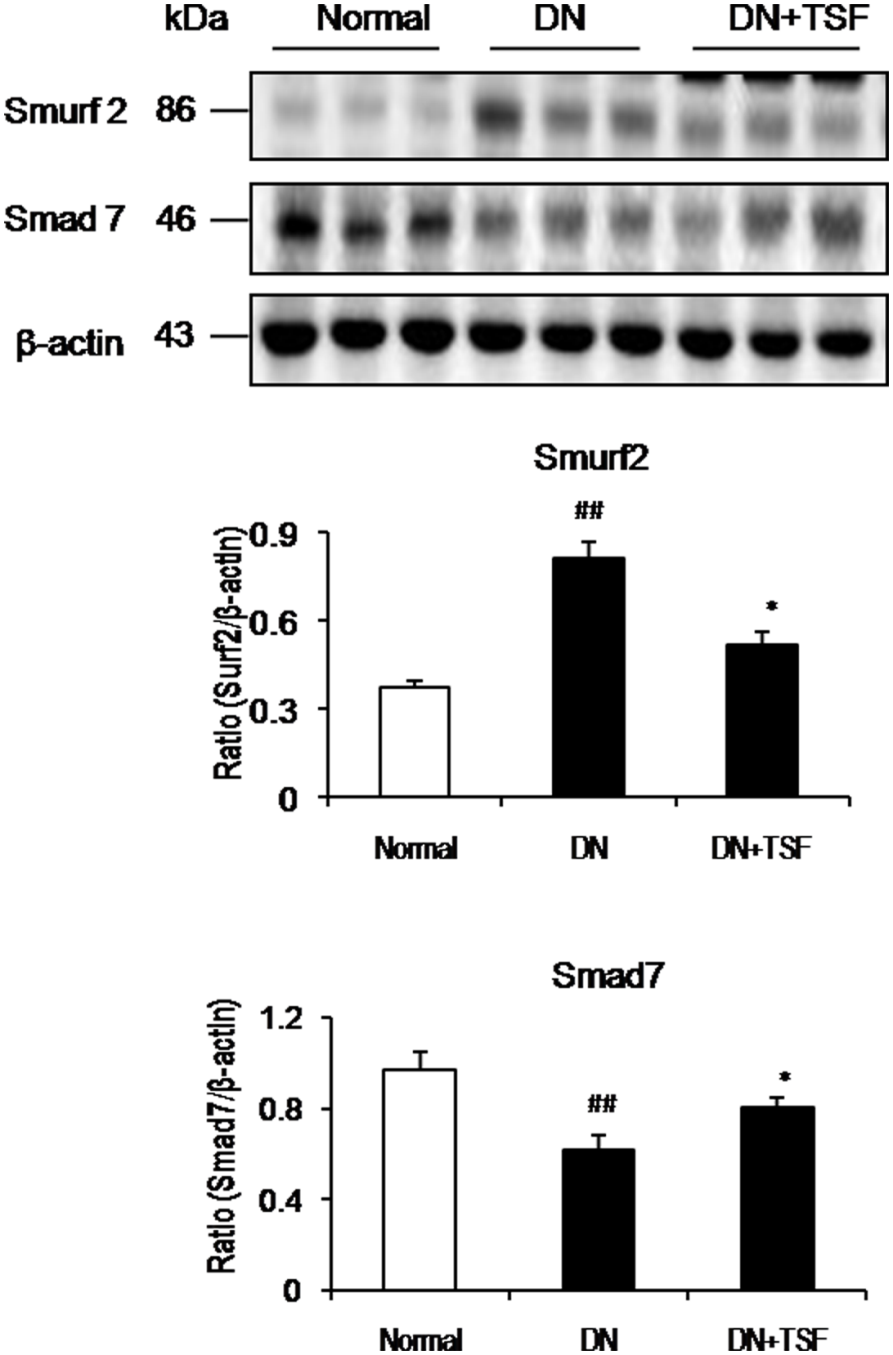
TSF regulates expression of renal Smurf2 and Smad7 in DN rats. Western blot and semi-quantitative analysis of renal Smurf2 and Smad7 show that TSF treatment largely reduces renal Smurf2 expression in diabetic rats, resulting in less degradation of Smad7 protein. Data are expressed as means ± SE for each group of 9rats. *P<0.05, **P< 0.01 TSF-treated group vs. DN group; ^#^*P*<0.05, ^##^*P*<0.01, ^###^*P*<0.001 DN group vs. normal group.

We have previously shown that Smad7 is an integrated regulator that negatively regulates TGF-β/Smad-mediated renal fibrosis via its negative feedback-loop and NF-κB-dependent renal inflammation by inducing IκBα, an inhibitor of NF-κB [12, 23]. However, Smad7 is degraded via a Smurf2-dependent proteasomal ubiquitin degradation mechanism [11]. Therefore, we examined whether TSF treatment alters the expression of Smurf2 and/or Smad7 in the diabetic kidney. Western blot analysis detected that Smurf2 expression was significantly increased in the diabetic kidney, which was associated with the reduction of Smad7 protein (Fig 7). In contrast, treatment with TSF reversed these changes by inhibiting Smurf2 and therefore protecting Smad7 from degradation (Fig 7).

## Discussion

The present study provided evidence that the Chinese herbal medicine Tangshen Formula (TSF) significantly inhibited albuminuria and largely alleviated diabetic kidney disease in a rat model of type 2 diabetes induced by high-fat diet and STZ injection. The therapeutic effect of TSF on diabetic kidney injury was associated with the inhibition of NF-κB-driven inflammation and TGF-β/Smad3-mediated renal fibrosis.

In this study, we successfully replicated the diabetic nephropathy in a type-2 diabetic rat model induced by high-fat diet-fed and low dose of streptozotocin. In comparison to normal rats, blood glucose levels and proteinuria in DN rats were increased significantly throughout the experiment, accompanied by the typical syndrome of diabetes such as polyphagia, polydipsia, polyuria and emaciation. Glomerular hypertrophy, mesangial matrix accumulation, and tubular atrophy were developed in DN rats and were inhibited after 20-week treatment with TSF. Interestingly, compared to the normal rats, the development of lower levels of serum creatinine in diabetic rats with or without TSF treatment may be attributed to the glomerular hyperfiltration occurred in the early stage of DN.

Renal fibrosis is a hallmark of chronic kidney disease including diabetic nephropathy and is mediated by TGF-β/Smad3 signaling [12, 25, 26]. A significant finding from the present study was that TSF protected kidneys from diabetic injuries by down-regulating expression of TGF-β1and TGF-β receptor I, thereby inactivating Smad3 signaling and blocking renal fibrosis including expression and accumulation of collagen I, IV, and fibronectin in the diabetic kidney. These findings were consistent with previous reports that mice lacking Smad3 are protected against renal fibrosis in a number of experimental models of chronic kidney diseases including hypertensive nephropathy and obstructive nephropathy [26–28]. Thus, it is possible that the renal protective effect of TSF on diabetes may be associated with the inhibition of TGF-β/Smad3-mediated renal fibrosis.

Inhibition of NF-B-driven renal inflammation may be another mechanism associated with the renoprotective effect of TSF on DN. It is now recognized that NF-κB is a central signaling pathway in inflammation. Many inflammatory mediators such as IL-1β, TNFα, MCP-1, can activate the NF-κB signaling pathway to mediate renal inflammation, resulting in progressive DN [27]. In the present study, treatment with TSF was capable of preventing IκBα (an inhibitor of NF-κB) from degradation, thereby inhibiting the activation of NF-κB signaling and NF-κB-driven renal inflammation in DN induced by high-fat diet and STZ injection. These findings suggest that TSF may negatively regulate NF-κB signaling to control renal inflammation in DN.

More significantly, we also found that inhibition of Smurf2-mediated ubiquitin degradation of Smad7 may be a central mechanism by which TSF suppressed both TGF-β/Smad3-mediated renal fibrosis and NF-κB-dependent renal inflammation in the diabetic kidney. In general, ubiquitination is a process that regulates ubiquitin molecules to attach to specific target proteins through the activation of three specific enzymes, including ubiquitin-activation enzyme (E1), ubiquitin-conjugation enzyme (E2), and ubiquitin ligase enzyme (E3) [28]. These polyubiquitinated target proteins are then disrupted and degraded by the 26S proteasome complex. Smurf2 has been reported to be an important E3 ligase that participates in modulating TGF-β-mediated signaling by targeting TGF-β receptor-1 and Smad2 (14, 15, and 32). Smad7 acts as an adaptor protein to help Smurf2 conjugating to TGF-β receptor-1 to cause its degradation, resulting in down-regulation of TGF-β signaling [11]. At the same time, ubiquitin-degradation of Smad7 occurs simultaneously [29]. If Smad7 is extensively degraded, Smad3 becomes overactivated and renal fibrosis is enhanced [30]. In addition, Smad7 is also a negative regulator of NF-κB signaling in both obstructive and diabetic nephropathy [31]. Smad7 can induce IκBα expression and prevents IκBα from degradation, thereby inactivating NF-κB signaling and suppressing NF-κB-dependent renal inflammation in *vivo* and in *vitro* [32, 33]. In this study, we clearly demonstrated that treatment with TSF resulted in a marked inhibition of Smurf2 and therefore blocked the Smurf2-mediated Smad7 ubiquitin degradation pathway. As the consequence of intact Smad7, activation of both TGF-β/Smad3 signaling and NF-κB signaling in the diabetic kidney was inhibited, thereby resulting in attenuation of renal fibrosis and inflammation. Thus, protection of Smad7 from degradation may be a key mechanism by which TSF inhibited diabetic kidney disease.

## Conclusions

In conclusion, the present study demonstrates that orally administered TSF significantly inhibits diabetic kidney disease. We also identify that prevention of Smurf2-mediated Smad7 ubiquitin degradation, thereby inhibiting both TGF-β/Smad3-mediated renal fibrosis and NF-κB-dependent renal inflammation may be mechanisms associated with the beneficial effect of TSF on DN.

## Supporting Information

S1 Fig Effect of TSF treatment on body weight (A), urine volume (B), and serum creatinine (C) in DN rats induced by high-fat-diet and STZ-injection. Data are expressed as means ±SE for each group of 9 rats. *P<0.05, **P<0.01 TSF-treated group vs. DN group; ^#^*P*<0.05, ^##^*P*<0.01, ^###^*P*<0.001 DN group vs. normal group.
(TIF)
