# Supplementary figures and images for: Therapeutic Effects of Tangshen Formula on Diabetic Nephropathy in Rats
Source: PLoS One. 2016 Jan 25;11(1):e0147693. doi: 10.1371/journal.pone.0147693 (PMC4726711; doi:10.1371/journal.pone.0147693)

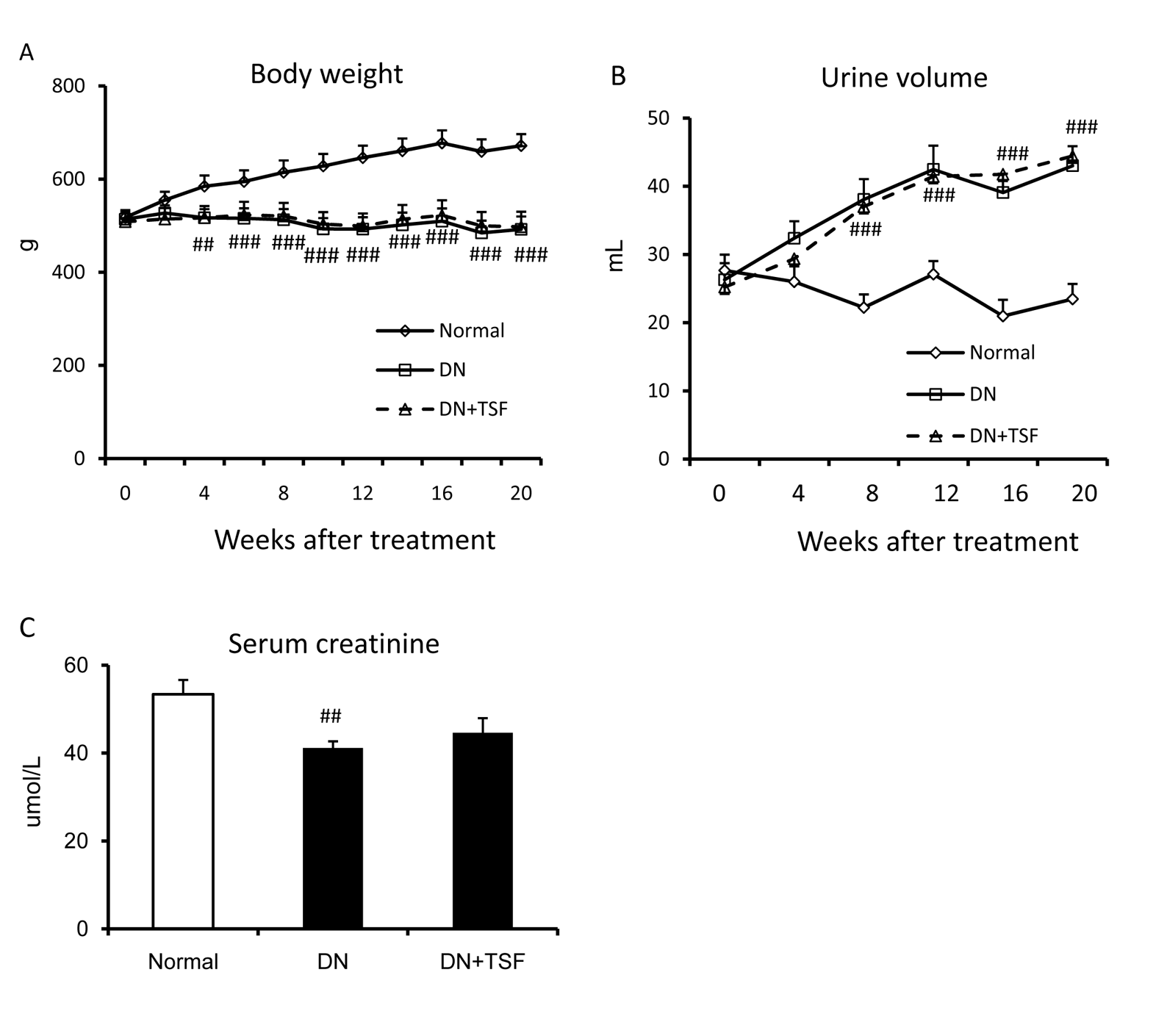

Supplement: S1 Fig — Data are expressed as means ±SE for each group of 9 rats. *P<0.05, **P<0.01 TSF-treated group vs. DN group; #P<0.05, ##P<0.01, ###P<0.001 DN group vs. normal group. (TIF) [file pone.0147693.s001.tif]
